# A Novel Classification Indicator of Type 1 and Type 2 Diabetes in China

**DOI:** 10.1038/s41598-017-17433-8

**Published:** 2017-12-12

**Authors:** Yannian Wang, Shanshan Liu, Ruoxi Chen, Zhongning Chen, Jinlei Yuan, Quanzhong Li

**Affiliations:** 1 School of Information Engineering, Zhengzhou University, 450001 Zhengzhou, Henan P.R. China; 2 Department of Cardiology, University of Arkansas for Medical Sciences, 72205 Little Rock, AR USA; 3 The Fifth Affiliated Hospital, Zhengzhou University, 450052 Zhengzhou, Henan P.R. China; 4 Department of Endocrinology, People’s Hospital of Zhengzhou University, 450003 Zhengzhou, Henan P.R. China

## Abstract

Because of the differences of treatment, it is extremely important to classify the types of diabetes, especially for the diagnosis made by clinician. In this study, we proposed a novel scheme calculating an indicator of classifying diabetes, which contains two stages: the first is a model of feature extraction, 17 features are automatically extracted from the curve of glucose concentration acquired by continuous glucose monitoring system (CGM); the second is a model of diabetes parameter regression based on an ensemble learning algorithm named double-Class AdaBoost. 1050 curves of glucose concentration of type 1 and type 2 diabetics were acquired at the Department of Endocrinology in People’s Hospital of Zhengzhou University China, and an upper threshold *μ* was set to 7 mmol/L, 8 mmol/L, 9 mmol/L, 10 mmo/L, and 11 mmol/L respectively according to the guideline of WHO. The experiments show that the coincidence rate of our scheme and clinical diagnosis is 90.3%. The novel indicator extends the criteria in diagnosing types of diabetes and provides doctors with a scalar to classify diabetes of type 1 and type 2.

## Introduction

Diabetes mellitus (DM) is a chronic metabolic disease caused by deficiency or diminished effectiveness of endogenous insulin. Poor glucose control can lead to complications in multiple organs resulting in increased rates of morbidity and mortality^1^. According to International Diabetes Federation (IDF), in the world, there are about 415 million patients suffering from diabetes in 2015^2^, and this number is growing.

The efficacy of treatment in preventing diabetes complications has been confirmed by Diabetes Control and Complications Trial^3^. Specifically, the success^4,5^ in Continuous Glucose Monitoring system (CGM), an invasive device is used to measure and record patients’ glucose concentration every 5 minutes. Recently, the CGM has been introduced in the prediction of glucose concentration^6,7^. We consider it is worth investigating the classification according to CGM signal as tools for the management of DM^8,9^.

According to the pathogeny of diabetes, there are 4 types of DM. Type 1 diabetes and Type 2 diabetes are the main categories. Clinically, the type is usually determined by tests, such as fasting plasma insulin (FINS), insulin releasing test (INS), C-Peptide test, insulin autoantibodies (IAA) and islet cell autoantibodies (ICA). Some of the tests are only temporary and incomplete to diagnose diabetes for the limitation of cognition. The scheme proposed in this paper tries to provide an effective and a supplementary indicator for diabetes classification, which would have benefits of perfecting the framework, raising the precision, and offering a convenient and intelligent method of classifying diabetes.

Classification is one of the hottest issues in data mining. Various classification algorithms have been introduced in many fields, such as Sound recognition^10^, Bitcoin fraud^11^, and Tomato plant disease^12^. In this study, a novel scheme calculating the indicator of classifying diabetes is proposed and it consists of two stages: the first is feature extraction, in which 17 features^13^ are automatically extracted from the curves of glucose concentration using statistics methods, and the second is diabetes parameter regression based on an ensemble learning algorithm named double-Class AdaBoost. The scheme can give an intelligent and precise method to diagnose the type of diabetes.

### The Scheme

Based on Adaboost and its variant, a diabetes classification indicator is proposed, and its processing steps are described below:

1. Utilize CGM, collect curves of diabetic glucose concentration.
2. Employ feature extraction model to achieve 17 features from the training curves of glucose concentration.
3. Build and train a classifier using the 17 features based on variants of AdaBoost.
4. Verify the classifier using the testing curves of glucose concentration.
5. Evaluate the indicator of the scheme to classify diabetes.

## Methods

### CGM

CGM is used in examination of how the blood glucose concentration reacts to insulin, exercise, food, and others. And it needs calibrating with traditional finger-stick measurements. A CGM acquires glucose concentration of patients on a continuous basis (every five minutes).

### Feature Extraction

**Figure 1:**
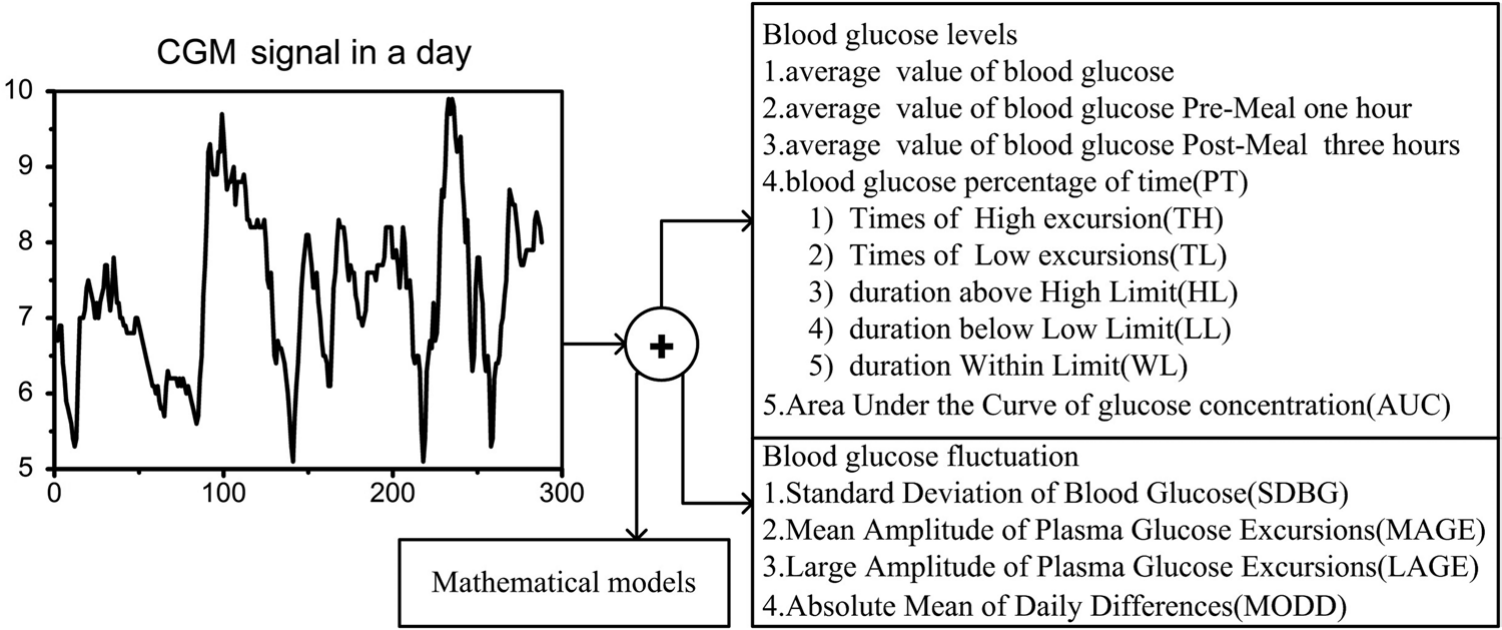
Feature extraction from CGM signal. The 17 features of blood glucose concentration and glucose fluctuation are extracted when CGM signal of one day is inputted to the Mathematical models.

Feature extraction^14^ is based on the morphological characteristics of signals to obtain the intrinsic features. Features usually possess some physical significance and could be extracted from complicated multi-component signals such as a time-series of glucose concentration. Hence, the feature extraction is taking a glucose concentration signal as input and gives the features as the output. The model of features extraction is illustrated in Figure 1.

The first feature is the average of blood glucose on the whole day; it can be calculated by Equation (1).

1 $$\overline{x}=\frac{1}{n}\sum _{i=1}^{n}{x}_{i}$$

where *x*_*i*_ is a discrete value of blood glucose concentration, *n* is the number of *x*_*i*_ in a day. The subsequent six features are also the average in different periods including pre-meal average and post-meal average of three meals. All those averages can be calculated by Equation (1).

SDBE this feature is Standard Deviation of Blood Glucose; it can be calculated by Equation (2).

2 $$SDBE=\sqrt{\frac{1}{n}\sum _{i=1}^{n}{({x}_{i}-\overline{x})}^{2}}$$

LAGE this feature is Large Amplitude of plasma Glucose Excursions; it can be calculated by Equation (3).

3 $$LAGE={x}_{\max }-{x}_{\min }$$

where *x*_*max*_, *x*_*min*_ are the maximum and minimum values of blood glucose in a day.

MODD this feature is absolute Mean Of Daily Differences, and it can be calculated by Equation (4).

4 $$MODD=\frac{1}{287}{\sum }_{i=1}^{288}|{v}_{1}(i)-{v}_{2}(i)|$$

where *v*_1_, *v*_2_ are arrays of glucose concentration with 288 values of one day, and they are the glucose concentrations of same diabetic in different day respectively.

Area Under the Curve of glucose concentration (AUC) indicates the area parceled by the glucose concentration-time curve and the threshold (upper threshold or lower threshold). The AUC should be calculated with two areas including under the curve of glucose concentration and the upper threshold, over the curve of glucose concentration and the lower threshold.

Mean amplitude of plasma glucose excursions (MAGE). This feature has been studied by many papers^15^. The MAGE can be calculated as follows: Step 1. Get all extreme points in the signal; Step 2. Find the first valid extreme point whose absolute differences of both adjacent extreme points are greater than the Standard Deviation of the signal; Step 3. Accumulate all differences of valid extreme point according to the left direction of the first valid extreme point in step 2; Step 4. MAGE is the average of sum counted in step 3.

5 $$MAGE=\frac{1}{n}\sum _{i=1,3,5,\mathrm{...}}^{2n-1}{ep}_{i}-{ep}_{i+1}$$

where *ep*_*i*_ is the left adjacent extreme point of valid extreme point *ep*_*i+1*_; *n* is the number of valid extreme point.

Blood glucose Percentage of Time (PT) includes two main aspects: times and the percentage of the time. The features relating to times, including Times of High excursion (TH), Times of Low excursion (TL), are the number of the extreme points of glucose concentration curve over threshold line in one day. The features relating to percentage of the time, including duration above High Limit (HL), duration below Low Limit (LL), duration Within Limit (WL), are the percentage of the time of glucose concentration curve over the threshold line one day.

In our research, a dataset was built to store the features, as shown in Table 1.

**Table 1:** The features dataset (mmol/L).

***Average***	***Pre-meal***			***PT***				
	***Breakfast***	***Lunch***	***Dinner***	***TH***	***TL***	***HL***	***LL***	***WL***
*6*.*7*	*6*.*6*	*7*.*9*	*5*.*7*	0	0	0	0	1
7.2	5.3	7.7	7.9	1	0.07	1	0.02	0.91
9.6	9.7	10.7	9.5	3	0.25	0	0	0.75
***AUC***	***SDBG***	***MAGE***	***LAGE***	***MODD***		***Post-meal***		
						***Breakfast***	***Lunch***	***Dinner***
0	*0*.*7*	*0*.8	*3*.*4*	*0*.*725*		*6*.*6*	*7*.*3*	*6*.*4*
*0*.*1*	*1*.*8*	*−2*.*6*	*8*.*4*	*1*.*4327*		*9*.*8*	*7*.*3*	*8*.*9*
*0*.*5*	*1*.*6*	*−5*.*05*	*8*	*2*.*7753*		10	*12*.*5*	*8*.*6*

Each row was extracted from a curve of blood glucose concentration of diabetics.

Usually, age is an important factor related to diabetes^16^, and places heavy weight on the classification of type of diabetes, thus would cause under-fitting. Some other factors were not involved, such as exercise, food, and insulin or oral medicines, which are difficult to quantify as these factors are from different manufacturers and are difficult to homogenize. Furthermore the main purpose of our research is to provide an easy and approach available to diabetes diagnose.

### AdaBoost

Boosting methods are iterative algorithms^17^. AdaBoost is a boosting method which united some simple “weak” classifiers to generate generalized models. It was proposed by Freund and Schapire to distinguish a binary classification^18^, and later various AdaBoost variants such as Real Adaboost were proposed^19^. AdaBoost and its variants have contributed to various real-world applications, such as face detection^20^ and human detection^21^. In our research, its variants Real Adaboost^19^, Gentle AdaBoost^22^, and Modest AdaBoost^23^, were applied to the model of diabetes parameter regression in our scheme.

### Diabetes parameter regression based on AdaBoost

Let s = {(g_1_,y_1_), (g_2_,y_2_), …, (g_m_,y_m_)} be a set of training samples with initial weights *D*_1_(*g*_*i*_) = 1/m, and m is the number of training data. Each *g*_*i*_ is a vector with 17 features which were extracted from CGM curve of glucose concentration, and each *y*_*i*_ is the label of *g*_*i*_. In our research, the DM classification is a binary classification, so assuming label *y*_*i*_ equals 1 if the sample belongs to type 1 diabetes, and otherwise equals −1 when the sample belongs to type 2 diabetes.

Diabetes classification based on AdaBoost algorithm is described as follows:

Input: training dataset s = {(g_1_,y_1_), (g_2_,y_2_), …, (g_m_,y_m_)}, initialize data weights *D*_1_(*g*_*i*_) = 1/m, i = 1, …, m;

$${{\rm{g}}}_{{\rm{i}}}\in {{\rm{G}}}^{17},{y}_{i}\in \{-1,1\}$$

$${\rm{For}}\,t=1,2,\ldots {\rm{T}}:$$

Step 1: train weak classifier *h*_*t*_ using distribution *D*_*t*_.

Step 2: Calculate the error of the weak classifier *h*_*t*_: G → {−1,1}.

$${\varepsilon }_{t}={\sum }_{i=1}^{m}{D}_{t}({g}_{i})[{y}_{i}\ne {h}_{j}({g}_{i})]$$

Step 3: Calculate the weight *a*_*t*_ = (1/2)*ln*((1 − *ε*_*t*_)/*ε*_*t*_).

Step 4: Update data weight *D*_*t*_ and get new weights *D*_*t*+*1*_ by error.

$${D}_{t+1}=\frac{{D}_{t}({g}_{i})\exp (-{a}_{t}{y}_{i}\,{h}_{t}({g}_{i}))}{{z}_{t}}$$

where z_t_ is a normalization factor.

Output: final classifier: ${H}_{final}(g)=sign({\sum }_{t=1}^{T}{a}_{t}{h}_{t}(g))$

### Diabetes parameter regression based on Variants algorithm of AdaBoost

Three variants algorithms of AdaBoost were used for diabetes parameter regression. Firstly, Real AdaBoost is a generalization of AdaBoost algorithm proposed by Schapire and Singer^19^. Its output is not binary, but a real number between + 1 and −1. Real AdaBoost algorithm seems to Adaboost, except the steps 1–3 summarized as below:

For each weak classifier *h*_*t*_

- every value space of features is divided into several disjoint blocks G_1_, …, G_n_
- under the distribution D_t_ calculate
  $${p}_{l}^{j}(g)=p({g}_{i}\in {G}_{j},{y}_{i}=l)={\sum }_{i=g={G}_{j},{y}_{i}=l}{D}_{t}(i)\,l\in 1,-1$$
- set the output of h on each G_j_ as
  $${h}_{t}^{j}(g)\leftarrow \frac{1}{2}\,{\rm{l}}{\rm{o}}{\rm{g}}\,{p}_{l}^{j}/(1-{p}_{l}^{j})\in R\quad {h}_{t}({g}_{i})={h}_{t}(g)$$
- calculate the normalization factor

$$z=2\sum _{j}\sqrt{{p}_{+1}^{j}{p}_{-1}^{j}}\quad {z}_{t}=\,{\rm{\min }}\,z\quad {h}_{t}={\rm{\arg }}\,{\rm{\min }}\,z$$

The second variants algorithm of AdaBoost named Modest AdaBoost which complete steps could be found in paper^23^. Gentle AdaBoost is the most efficient boosting algorithm and it has been used in Cascades object detection^24^. In each epoch, Gentle AdaBoost does a weighted regression based on least square. It means that the regression function h_*t*_(*g*) is fit by weighted least-squares of *y*_*i*_ to *g*_*i*_.

### Model Evaluation

In order to evaluate classification results, the present study applied two performance indicators: ACC (accuracy) and MCC (Matthews correlation coefficient). P and N represent the positive class and negative class respectively. T and F denote True and False respectively, as described in Table 2.

**Table 2:** Confusion matrix. In this table, TP is the number of true positives, TN the number of the true negatives, FP the number of false positives and FN the number of false negatives.

	Predict P	Predict N
True P	TP	FN
True N	FP	TN

The ACC is as the formula

6 $$ACC=\frac{TP+TN}{P+N}$$

The MCC is as the formula

7 $$MCC=\frac{TP\times TN-FP\times FN}{\sqrt{(TP+FP)(TP+FN)(TN+FP)(TN+FN)}}$$

## Results

### Patient Database

The diabetics were screened at ages ranging from 40 to 60 and the glucose concentration were acquired from the Department of Endocrinology in People’s Hospital of Henan Province of China. There are 1050 samples of diabetes glucose concentration, and each sample is a curve with more than 864 values.

### Experiment and analyses

To demonstrate the performance of proposed indicator, 300 of the 1050 samples were used as training set to construct a diabetes classifier while the other 750 were used as testing set to evaluate the classifier. Besides, 7 mmol/L, 8 mmol/L, 9 mmol/L, 10 mmol/L and 11 mmol/L were set to the upper threshold of glucose target range in the progress of feature extraction from the curves of glucose concentration monitored by CGM.

The Committee Report of diabetes expert of WHO diagnoses DM with fasting blood glucose concentration between 6.1 mmol/L and 6.9 mmol/L and plasma glucose of 11.1 mmol/L 2 hours post glucose-load (2 h PPG). This is the reason why 7 mmol/L, 8 mmol/L, 9 mmol/L, 10mmo/L and 11 mmol/L were selected as the upper threshold when we extract the 17 features from glucose signal.

The models of Real AdaBoost, Modest AdaBoost and Gentle AdaBoost were applied to calculating the indicator of classification diabetes and the error rate was presented in Table 3. The error rate of Modest AdaBoost is 0.0970 when the upper threshold was set at 7 mmol/L and 8 mmol/L, which means that the coincidence rate of our scheme and clinical diagnosis is 90.3%.

**Table 3:** Comparison of error rate.

Upper limit	7 mmol/L	8 mmol/L	9 mmol/L	10 mmol/L	11 mmol/L
Error rate					
Real AdaBoost	0.0921	0.0867	0.1491	0.1382	0.1057
Modest AdaBoost	0.0970	0.0970	0.1084	0.1220	0.1165
Gentle AdaBoost	0.0678	0.0786	0.1274	0.1030	0.1003

The error rate of Real AdaBoost, Modest AdaBoost, and Gentle AdaBoost at 7, 8, 9, 10 and 11 mmol/L of the upper threshold of glucose target range.

**Figure 2:**
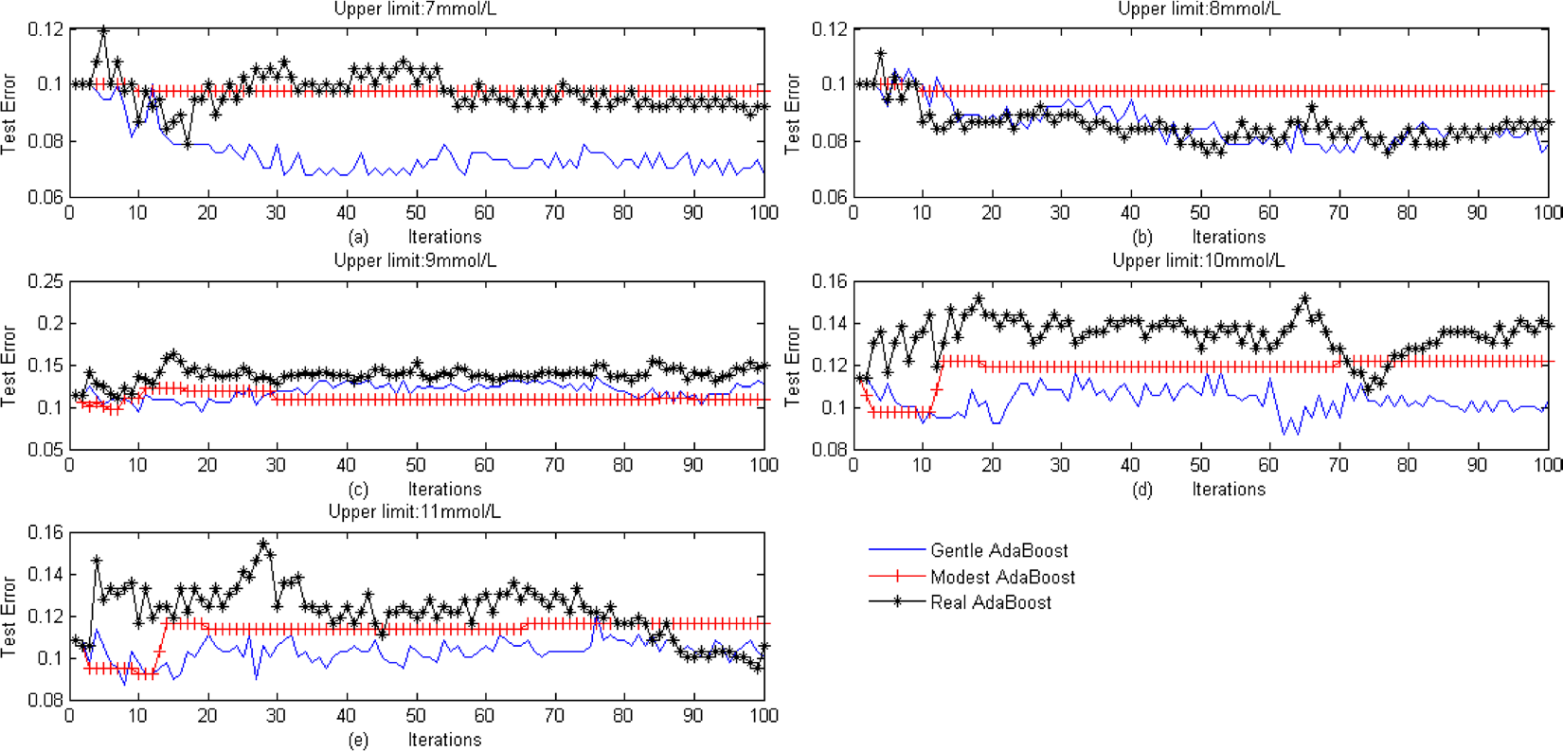
Comparison error of diabetes classification. Test error of the three ensemble methods, Real AdaBoost, Modest AdaBoost, Gentle AdaBoost, at 7, 8, 9, 10 and 11 mmol/L of the upper threshold of glucose target range. The *y*-coordinate of each point gives the test error rate, and the x-coordinate gives the times of iterations.

After training 100 iterations, the three models of Real AdaBoost, Modest AdaBoost, and Gentle AdaBoost were to calculate the indicator of classifying diabetes. The test misjudging rate of indicator and clinical diagnosis illustrated in Figure 2. The upper thresholds of Figure 2 (a)–(e) were set at 7, 8, 9, 10 and 11 mmol/L respectively. It shows that when the upper limit was set at 7 mmol/L and 8 mmol/L the misjudging rate of three models were lower, and the misjudging rate of Model AdaBoost depicted by the line with the mark ‘|’ is 0.0970. Furthermore, when the upper threshold was set at 10 mmol/L, the three models perform worst in diabetes classification. But when the upper threshold was set at 9 mmol/L or 11 mmol/L, the misjudging rate of Real AdaBoost is changing, and its largest error is greater than 0.12, therefore 9 mmol/L and 11 mmol/L are not suitable for regarding as the upper threshold. The value of upper threshold affects results of diabetes classification.

**Figure 3:**
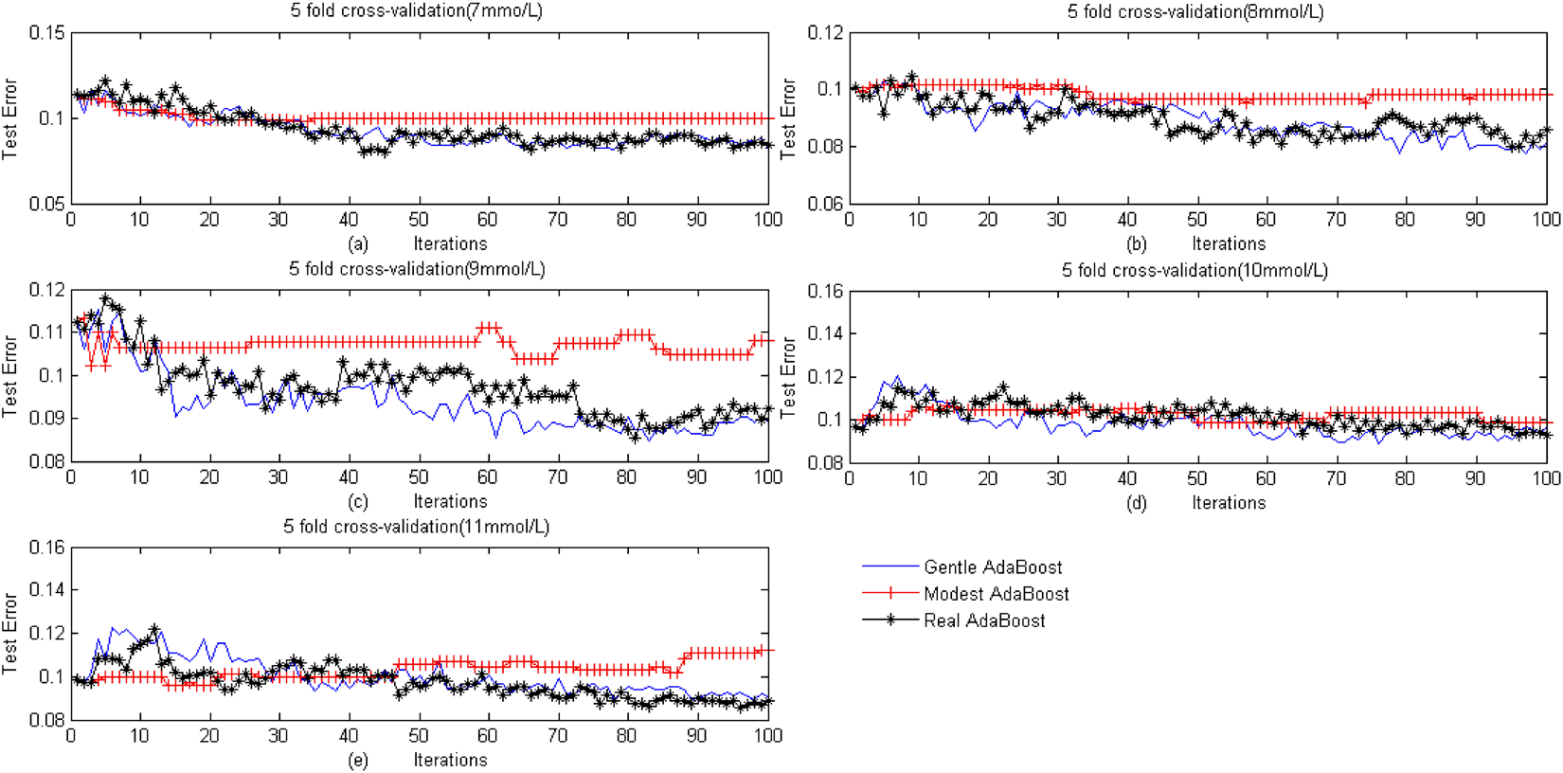
5-fold cross-validation. The method of 5 fold cross-validationto validates the test error of Real AdaBoost, Model AdaBoost and Gentle AdaBoost at 7, 8, 9, 10 and 11 mmol/L of the upper limit of glucose target range. The *y*-coordinate of each point gives the test error rate, and the x-coordinate gives the times of iterations.

5-fold cross-validations were used to further demonstrate the accuracy of our scheme and seek out the best of upper threshold, after training 100 iterations, the indicator of classifying diabetes based on Real AdaBoost, Modest AdaBoost and Gentle AdaBoost were calculated and the test misjudging rate of indicator and clinical diagnosis illustrated in Figure 3. It shows that when threshold was set at 7 mmol/L or 8 mmol/L, the performance of our scheme is better, and only a few misjudging rates were above 0.1. It indicates that the coincidence rate of indicator calculated by our scheme and clinical diagnosis is better and the indicator is useful for doctors to diagnose diabetes.

The performance of our scheme was evaluated when the threshold was set at 7, 8, 9, 10 and 11mmol/L respectively. The results are shown in Table 4. It shows that when threshold was set at 7 mmol/L or 8 mmol/L, the performance of our scheme is better.

**Table 4:** Comparison of accuracy and Matthews correlation coefficient.

Upper limit	7 mmol/L	8 mmol/L	9 mmol/L	10 mmol/L	11 mmol/L
ACC	0.9038	0.9038	0.8428	0.8293	0.8293
MCC	0.5103	0.4736	0.0890	0.0872	0.0662

The ACC and MCC of Modest AdaBoost at 7, 8, 9, 10 and 11 mmol/L of the upper threshold of glucose target range.

## Discussion

Due to the difference of epidemiology, etiology, pathogenesis and treatment of type 1 and type 2 DM, a knotty problem is how to effectively treat diabetes in clinic^25^. For a doctor, the reasonable solution is to classify the type of diabetes and suit the remedy to the case, so the diabetes can be in control. In fact, there are many clinical indicators to classify diabetes, such as the test results of Oral Glucose Tolerance Test (OGTT), INS, C-Peptide, IAA, ICA. The tests would contribute to providing guideline in treating diabetes, but the tests are incomplete and can’t precisely reflect the heterogeneity of the Type 1 diabetes and Type 2 diabetes. Moreover, some of original symptoms about Type 2 diabetes have emerged on patients with Type 1 diabetes. At the moment, CGM can monitor the curve representing the fluctuation of glucose concentration in patients with type 1 and 2 diabetes^9,10^, which is one of the most successful cases for diabetes controlling. In addition, the 17 features would be extracted from the curve of glucose concentration^13^. Those features can’t directly diagnose the type of DM, but we attempt to build a novel scheme calculating the indicator of classifying DM by using those features.

We have constructed an effective scheme, which consists of feature extraction and classification. The experimental results show when the upper threshold *μ* is correctly set, the misjudging rate of classification is less than 0.097, which suggests that the scheme achieves the best performance and the coincidence rate of our scheme and clinical diagnosis is up to 90.3%.

This experiment indicates that an indicator can be extracted from the curve of glucose concentration based on CGM and it is helpful for doctors to classify diabetes. In addition, more works should be considered, such as how to improve the precision of classifying diabetes, how to set a novel penalty to rectify the weight of diabetes samples according to the sampling distribution (D_t_) of diabetes in the process of iteration, and our scheme should be validated whether it suffers data imbalance problems^26,27^.
